# Genome-Wide Identification and Expression Analysis of MYB Transcription Factor Superfamily in *Dendrobium catenatum*

**DOI:** 10.3389/fgene.2021.714696

**Published:** 2021-08-26

**Authors:** Tingting Zhang, Zheng Cui, Yuxin Li, Yuqian Kang, Xiqiang Song, Jian Wang, Yang Zhou

**Affiliations:** ^1^Key Laboratory for Quality Regulation of Tropical Horticultural Crops of Hainan Province, School of Horticulture, Hainan University, Haikou, China; ^2^Key Laboratory of Ministry of Education for Genetics and Germplasm Innovation of Tropical Special Trees and Ornamental Plants, Key Laboratory of Germplasm Resources Biology of Tropical Special Ornamental Plants of Hainan Province, School of Forestry, Hainan University, Haikou, China

**Keywords:** *Dendrobium catenatum*, MYB transcription factors, gene family, gene expression, abiotic stress

## Abstract

*Dendrobium catenatum* is an important traditional Chinese medicine and naturally grows on tree trunks and cliffs, where it can encounter diverse environmental stimuli. MYB transcription factors are widely involved in response to abiotic stresses. However, the *MYB* gene family has not yet been systematically cataloged in *D. catenatum*. In this study, a total of 133 MYB proteins were identified in *D. catenatum*, including 32 MYB-related, 99 R2R3-MYB, 1 3R-MYB, and 1 4R-MYB proteins. Phylogenetic relationships, conserved motifs, gene structures, and expression profiles in response to abiotic stresses were then analyzed. Phylogenetic analysis revealed MYB proteins in *D. catenatum* could be divided into 14 subgroups, which was supported by the conserved motif compositions and gene structures. Differential *DcMYB* gene expression and specific responses were analyzed under drought, heat, cold, and salt stresses using RNA-seq and validated by qRT-PCR. Forty-two *MYB* genes were differentially screened following exposure to abiotic stresses. Five, 12, 11, and 14 genes were specifically expressed in response to drought, heat, cold, and salt stress, respectively. This study identified candidate *MYB* genes with possible roles in abiotic tolerance and established a theoretical foundation for molecular breeding of *D. catenatum*.

## Introduction

Transcription factors (TFs), also commonly called *trans*-acting factors, specifically bind to *cis*-acting elements of promoter regions in eukaryotic genes and then activate or inhibit target gene expression (Riechmann et al., 2000). TFs usually consist of four functional regions: a DNA-binding domain, transcriptional regulatory region, oligomerization site, and nuclear localization signal (De Vos et al., 2006). *MYB* TFs, which are part of one of the largest families of TFs in eukaryotic organisms, are named for the conserved MYB DNA-binding domains in their N-terminus (Lipsick, 1996; Chen et al., 2009; Dubos et al., 2010), which typically consist of 1–4 imperfect repeat (R) motifs (Du et al., 2009; Feller et al., 2011). Each repeat contains approximately 52 amino acids and is comprised of three helices, in which the second and third helices are separated by 3 conserved tryptophan residues with an interval of 18–19 amino acids, forming a helix-turn-helix (HTH) structure (Stracke et al., 2001). The HTH structure interacts with the major groove of DNA at the recognition site C/TAACG/TG and stabilizes the DNA-binding domain (Ogata et al., 1995). The C-terminus of MYB proteins are regulatory domains and highly divergent, which is consistent with the extensive range of regulatory roles for the *MYB* gene family (Jin and Martin, 1999).

All MYB TFs can be classified into one of four subgroups according to the number of their R motifs: MYB-related, R2R3-MYB, 3R-MYB, and 4R-MYB. MYB-related proteins contain a single or incomplete MYB repeat (Jiao et al., 2016) and play an important role in maintaining the integrity of chromosome structure and regulating gene transcription. The R2R3-MYB protein subgroup is the most important of the plant MYB family and contains the most members. R2R3-MYB proteins in plant species can be classified into 25 or more classes (Li and Lu, 2014; Feng et al., 2015; Salih et al., 2016). The genes of the R2R3-MYB subgroup may have evolved from 3R-MYB through the loss of an R1 repeat (Rosinski and Atchley, 1998). The third subgroup is 3R-MYB, i.e., R1R2R3-MYB, and members are composed of three continuous repeats, R1, R2, and R3, and are found in most eukaryotes. It has been hypothesized the 3R-MYB gene evolved from the R2R3-MYB gene through acquisition of the R1 motif (Jiang et al., 2004). 4R-MYB is the smallest type of plant MYB TF and contains four R1/R2 repeats (Zimmermann et al., 2004; Ito, 2005; Li and Lu, 2014).

Studies have shown MYB proteins play important roles in a variety of biological processes in plants, including in cell differentiation and morphogenesis, plant growth and development, and responses to stress (Chen et al., 2014). They have also been found to regulate pigment biosynthesis in many horticultural plants (Jiang et al., 2019; Meng et al., 2019; Xu et al., 2020), as well as plant florescence (Persak and Pitzschke, 2013). For example, **Arabidopsis** plants expressing mutant **myb44** fluoresced earlier than wild-type whenever under long-day or short-day. Conversely, the florescence of **Arabidopsis** plants overexpressing **AtMYB44** was significantly delayed compared to wild-type plants (Persak and Pitzschke, 2013). MYB proteins have been demonstrated to be involved in a number of abiotic stress responses. The 1R-MYB TF enhances drought tolerance in chickpea plants through interactions with GSGT2, CIPK25, and ABR17-like proteins (Ramalingam et al., 2015). Transgenic tomatoes overexpressing the R2R3-type MYB gene **SlMYB102** have higher salt tolerance than wild-type plants (Zhang X. et al., 2020). In sweet potato, the R2R3-MYB gene **IbMYB116** enhances drought tolerance through the JA signaling pathway (Zhou Y. et al., 2019). Soybean **GmMYB84** is induced by salinity in a DNA demethylation-dependent manner and confers salt tolerance in transgenic **Arabidopsis** (Zhang W. et al., 2020). MdMYB23 enhances the cold tolerance of transgenic apple calli and **Arabidopsis** (An et al., 2018). In wheat, several **MYB** genes have been identified that are involved in increased tolerance to salt, drought, and high temperatures (Qin et al., 2012; Zhang et al., 2014; Zhao et al., 2017). MYB proteins are also involved in responses to biotic stresses. The 1R-MYB TF LHY plays key roles in resistance to stripe rust in wheat (Zhang et al., 2015), while **Chrysanthemun** MYB15 might enhance aphid resistance by regulating lignin accumulation (An et al., 2019).

*Dendrobium catenatum* is a plant with great economic value as a traditional Chinese medicine. *D. catenatum* is a typical epiphytic orchid flower and grows on damp rocks in mountain climates and tree trunks in primeval forests in warm and humid environments. Abiotic stresses, including drought, high and low temperatures, and salt, seriously threaten the normal growth of *D. catenatum*. Therefore, it is necessary to identify and study the functions of stress-related genes in *D. catenatum*. In this study, we performed genome-wide identification of *MYB* genes based on the full genomic sequence of *D. catenatum* (Zhang et al., 2016) and investigated their physicochemical properties, phylogenetic relationships, conserved domains, gene structures, and expression patterns in response to abiotic stresses. Our study lays a foundation for future research into the functions of *D. catenatum MYB (DcMYB)* genes.

## Materials and Methods

### Identification of MYB Genes in *Dendrobium catenatum*

The annotated genomic sequence of *D. catenatum* (PRJNA262478) was downloaded from GenBank at NCBI (Zhang et al., 2016). To identify *D. catenatum* MYB genes, the Hidden Markov Model (HMM) profile of MYB_DNA-binding (PF00249) was downloaded from the Pfam database^1^ and used as a query to search the *D. catenatum* protein database through the Bio-linux bioinformatics documentation system. All output proteins with an E value ≤ 1e-10 (Salih et al., 2016) were collected and conserved MYB domains were examined using Pfam, CDD^2^ and the SMART^3^ database. After excluding redundant sequences lacking the common MYB domain and incomplete sequences, candidate DcMYB proteins were confirmed. The locus of each gene in the scaffold and the isoelectric point (pI) and molecular weight (MW) of each putative MYB protein were analyzed using the Bio-linux system. The number of transmembrane regions was determined using TMHMM software.^4^ The subcellular localization of MYB proteins was predicted using the PSORT tool.^5^

### Phylogenetic Analysis of MYB Proteins

To study the phylogenetic relationships of MYB proteins, multiple alignments of the conserved domains of MYB proteins from *D. catenatum*, *Arabidopsis thaliana*, and *Oryza sativa* were performed using clustalw 2.0 and the results were used to construct a phylogenetic tree by the Maximum Likelihood method with MEGA-X. The bootstrap test method was adopted and replicates set to 1000. The phylogenetic tree was visualized and enhanced using the EvolView online tool.^6^

### Detection of MYB Protein Conserved Motifs

The conserved motifs in the DcMYB protein sequences were analyzed in Bio-linux with the following parameters: maximum number of motifs was 10, minimum motif width was 6, and maximum motif width was 100. The result was visualized using TBtools (Chen et al., 2020). To analyze the sequence features of MYB repeats, the amino acid sequences of R2 and R3 repeats in R2R3 MYB proteins in *D. catenatum* were extracted and underwent multiple sequence alignments using clustalw 2.0. The sequence logos for R2 and R3 repeats were generated using WebLogo^7^ with default settings (Zhou Q. et al., 2019).

### Gene Structure and Promoter *cis*-Regulatory Element Analysis

The *MYB* gene exon and intron information was extracted from the *D. catenatum* genome general feature format file using the Bio-linux system and then structures were visualized with TBtools. To identify potential *cis*-elements in the promoters of *MYB* genes, 2000-bp upstream sequences of the coding regions of the *DcMYB* genes were first obtained using the Bio-linux system, and then analyzed through the PlantCARE database.^8^

### Plant Materials and Stress Treatments

*D. catenatum* ‘Guangnan’ tissue culture seedlings were grown under a 12 h/25°C day and 12 h/22°C night regime with a relative humidity of 70% in a growth chamber at Hainan University. Three-month-old plantlets with uniform and robust growth were then selected for subsequent experiments. The seedlings were treated with 20% PEG8000 and 200 mM NaCl to simulate drought and salt stress, respectively. For temperature stress, the plantlets were transferred to a growth chamber under 42 and 4°C conditions for heat and cold stress, respectively. The seedlings were then collected and frozen in liquid nitrogen at different time points (0, 9, and 24 h) after treatment.

To analyze the tissue specificity of the *MYB* genes, a number of tissues, including roots, stems, leaves, sepals, capsules, and gynostemia, were collected from mature plants and frozen in liquid nitrogen.

### Gene Expression Analysis in Different Tissues

Total RNA was extracted from different tissues using a RNAprep Pure Plant kit (Tiangen Biotech (Beijing) Co., Ltd, China, DP441) according to the manufacturer’s handbook and concentrations were measured using a NanoDrop (Thermo, ND-2000). Equivalent amounts of RNA was reverse-transcribed into cDNA using the PrimeScript^TM^ RT reagent kit with gDNA Eraser (TaKaRa, Japan, RR047A) according to the manufacturer’s instructions. Ten genes across four subgroups were selected randomly for analysis to study their tissue-specific profiles. Quantitative real-time PCR (qRT-PCR) was performed using an Applied Biosystems QuantStudio 3 & 5 system (ThermoFisher Scientific, United States). The qRT-PCR amplification conditions were as follows: 95°C for 1 min, followed by 45 cycles at 95°C for 5 s and 60°C for 30 s. A dissociation curve from 60 to 95°C was generated to verify primer specificity. The relative expression levels were calculated by the 2^–△△CT^ method. The housekeeping gene of *Actin* was used as an internal control. Three replicate biological experiments were conducted. The results were visualized as a heat map constructed with TBtools (Chen et al., 2020). All primers are designed to avoid the conserved region and listed in Supplementary Table 2.

### Illumina Sequencing and Expression Levels of *MYB* Genes

Samples collected after exposure to drought, heat, cold, and salt stresses for 0, 9, and 24 h were analyzed by RNA-seq by Sangon Biotech (Shanghai, China). The following detailed experiment was provided by the company. According to the previous studies (Lei et al., 2018; Liu Y. et al., 2020), gene expression levels were calculated and normalized to the FPKM (fragments per kilobase of exon model per million mapped reads) values, where FPKM was equal to total exon fragments divided by the million mapped reads and kilobase of transcript. Then the expression data for *MYB* genes following exposure to different stresses was extracted in RStudio version 1.2.5019. Heat maps of differential expression of *MYB* genes were generated using RStudio.

### Quantitative Real-Time PCR Validation

To validate the accuracy of the RNA-seq assay, six genes were randomly selected to study their relative expression in response to abiotic stresses by qRT-PCR as described above. The gene-specific primers were designed using Primer Premier 5 software and are listed in Supplementary Table 2.

## Results

### Genome-Wide Identification and Classification of *MYB* Genes in *Dendrobium catenatum*

To identify MYB proteins in *D. catenatum*, the genomic sequence was downloaded from NCBI.^9^ After searching through the Bio-linux system using PF00249 as a seed sequence to perform HMMER alignment and removing redundant proteins and incomplete sequences, 133 candidate *MYB* genes were identified and designated *DcMYB1* to *DcMYB133* (Supplementary Table 1). These MYB TFs were classified into four distinct groups: MYB-related family (32 proteins, 24.06%), R2R3-MYB family (99 proteins, 74.44%), 3R-MYB (1 protein, 0.75%), and 4R-MYB families (1 protein, 0.75%), where the R2R3-MYB family contained the most *DcMYB* genes (Supplementary Table 3). The characteristics of each gene, including gene locus, protein length, MW, pI, number of transmembrane regions, and subcellular localization of MYB proteins, are listed in Supplementary Table 1. The proteins encoded by these genes ranged from 85 (DcMYB41) to 903 amino acids (DcMYB118), with an average number of 305 amino acids. The MWs of the predicted MYB proteins ranged from 10.19 kDa in DcMYB41 to 102.90 kDa in DcMYB118. Additionally, the theoretical pI averaged 7.48 (4.18 to 10.63; Supplementary Table 3). All of the proteins lacked transmembrane domains, except DcMYB100, which contained two. We also predicted the subcellular localizations of these MYB proteins using the PSORT tool. Most of the proteins were predicted to be nuclear, except DcMYB41, DcMYB58, and DcMYB85 were cytoplasmic, DcMYB45, DcMYB78, and DcMYB117 were mitochondrial, and DcMYB100 was in the endoplasmic reticulum. These results suggest there are significant differences among the MYB proteins, which may reflect a diversity of functions in *D. catenatum*.

### Phylogenetic Analysis of DcMYB Proteins

**FIGURE 1:**
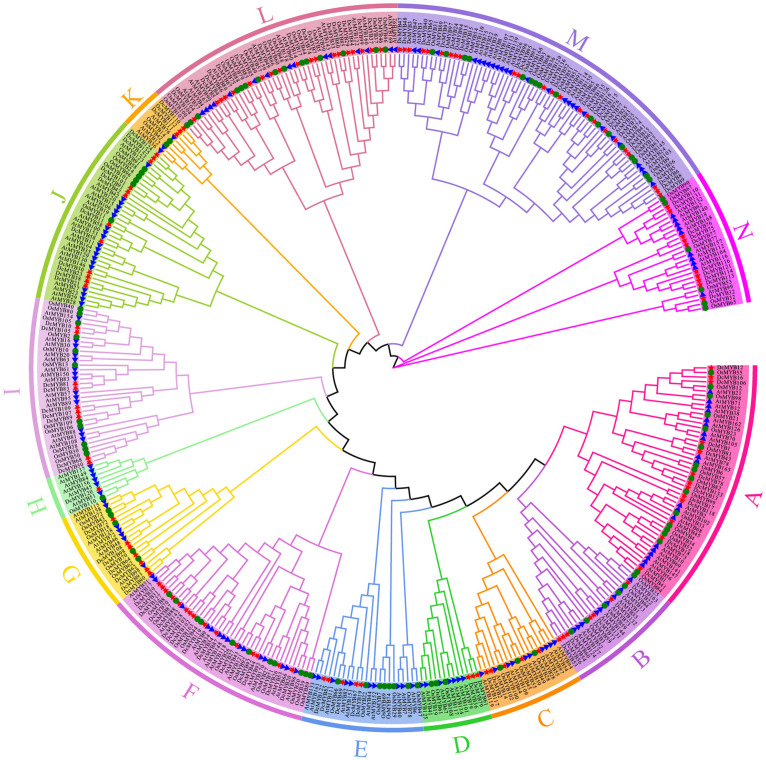
Phylogenetic analyses of MYB proteins. A phylogenetic tree of MYB proteins was constructed using MEGA-X with default parameters. The 14 subgroups are indicated with different colors. The red stars represent *Dendrobium catenatum* MYBs (DcMYBs), the blue triangles represent *Arabidopsis thaliana* MYBs (AtMYBs), and the green circles represent *Oryza sativa* MYBs (OsMYBs).

In this study, the phylogenetic relationship of *D. catenatum* MYB was determined using the Maximum Likelihood method with 1000 bootstrap replicates to construct an unrooted phylogenetic tree based on the alignments of 170 *A. thaliana* MYB (AtMYB) proteins and 110 *Oryza sativa* MYB (OsMYB) proteins (Supplementary Table 4). Based on their similarity between AtMYBs and OsMYBs, *D. catenatum* MYB proteins were named DcMYB1 to DcMYB133 (Supplementary Table 1). Phylogenetic analysis shows DcMYB family proteins can be divided into 14 subgroups (Figure 1) with each subgroup containing a different proportion of members (Supplementary Figure 1). The subgroup M contained 24 genes and had the most members (18.05%), followed by subgroups L (14.29%), F (11.28%), A (9.77%), J (9.02%), N (7.52%), I (6.77%), B (5.26%), G (5.26%), C (3.76%), E (3.76%), D (2.26%), K (2.26%), and H (0.75%), where H only contained 1 member. As seen in Figure 1 and Supplementary Figure 1, a similar member distribution in each subgroup was also found in *Arabidopsis* and rice.

### Conserved Motifs and Gene Structures of MYB Family in *Dendrobium catenatum*

**FIGURE 2:**
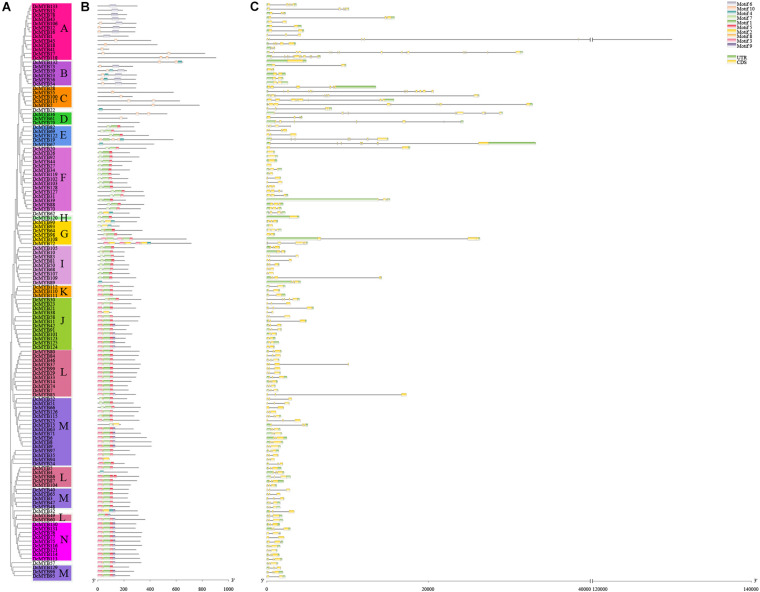
Phylogenetic relationships, structures, and motifs of DcMYB family members. **(A)** A phylogenetic tree of 133 DcMYB proteins was constructed with the Maximum Likelihood method. The different subgroups are indicated with different background colors and letters. **(B)** Conserved motifs of DcMYB proteins. Different motifs are represented by various colored boxes. **(C)** Exon/intron structures of *DcMYB* genes. Exon(s), intron(s), and UTR(s) are represented by yellow boxes, black lines, and green boxes, respectively. The phylogenetic tree, conserved motifs, and gene structures were predicted with TBtools.

**FIGURE 3:**
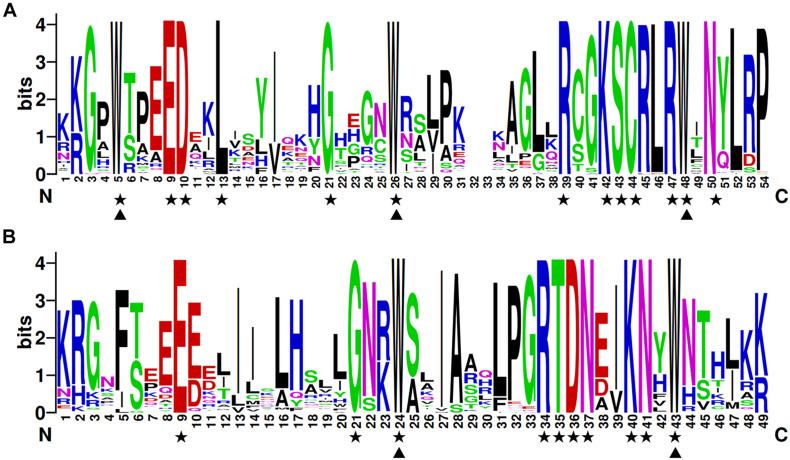
The R2 and R3 MYB repeats are highly conserved across all R2R3-MYB proteins in *D. catenatum*. The sequence logos of the R2 **(A)** and R3 **(B)** MYB repeats are based on full-length alignments of all the *D. catenatum* R2R3-MYB domains. The bit score indicates the information content for each position in the sequence. Triangles indicate conserved tryptophan residues (Trp) in the MYB domain and pentacles denote conserved residues that are identical among all R2R3-MYBs.

**FIGURE 4:**
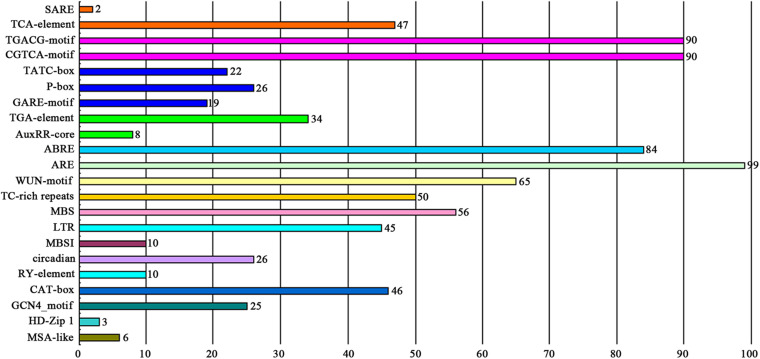
The number of DcMYB promoters containing various *cis*-acting elements. Different colors represent different *cis*-element types. The numbers above the columns represent the number of promoters.

**FIGURE 5:**
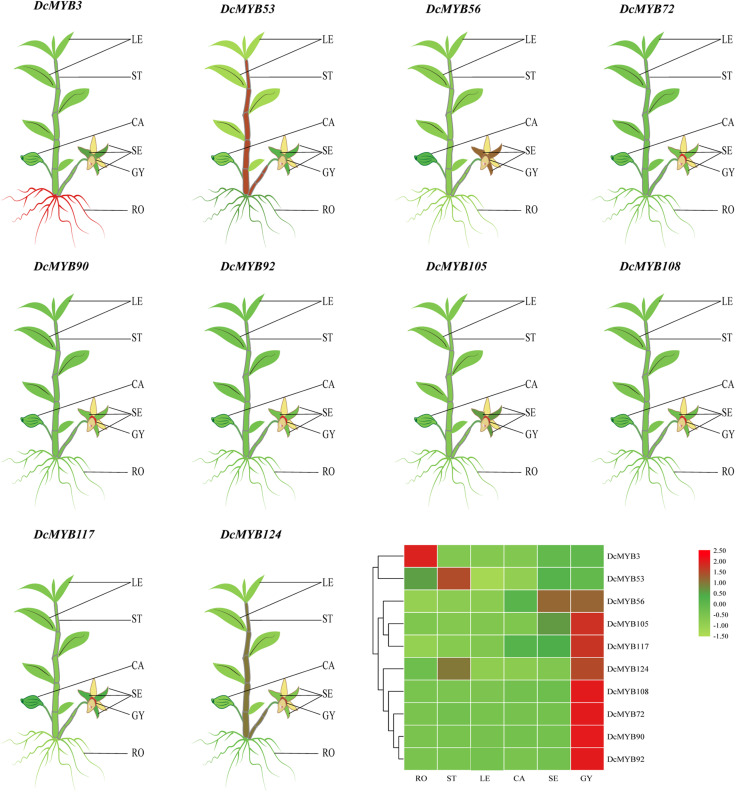
Tissue-specific expression of representative *DcMYB* genes by qRT-PCR. The mean expression value was calculated from three independent biological replicates relative to that in roots. The mean expression values were visualized by TBtools; red and green represent high and low expression levels, respectively. The raw data of the relative expression values and standard errors is provided in Supplementary Figure 3. *DcMYB* genes with similar profiles in the array were grouped on the left by a hierarchical clustering method. RO, root; ST, stem; LE, leaf; CA, capsule; SE, sepal; GY, gynostemia.

**FIGURE 6:**
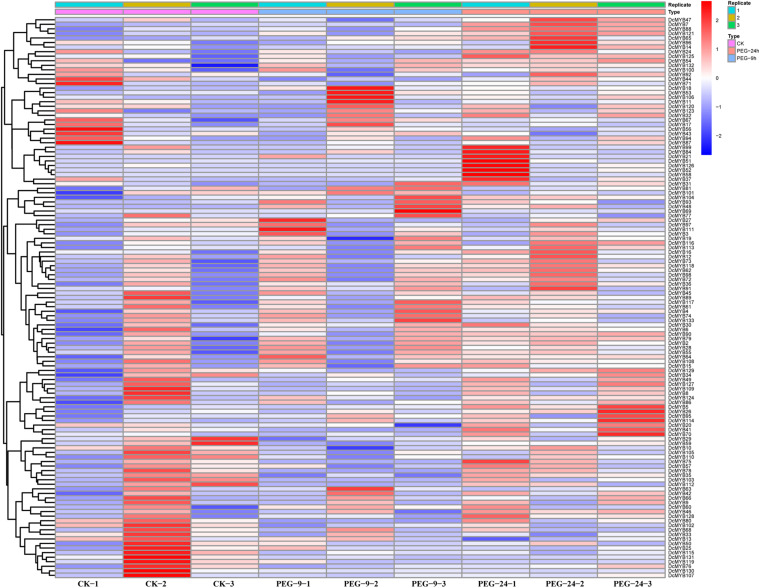
*DcMYB* gene expression in response to drought stress as determined by RNA-seq. Relative expression of different *MYBs* is shown for control and under drought stress after 0, 9, and 24 h. The *Y*-axis shows the gene names and *X*-axis represents the time interval. The different colors correspond to the log2-transformed fold change with blue and red indicating down- and up-regulation, respectively.

**FIGURE 7:**
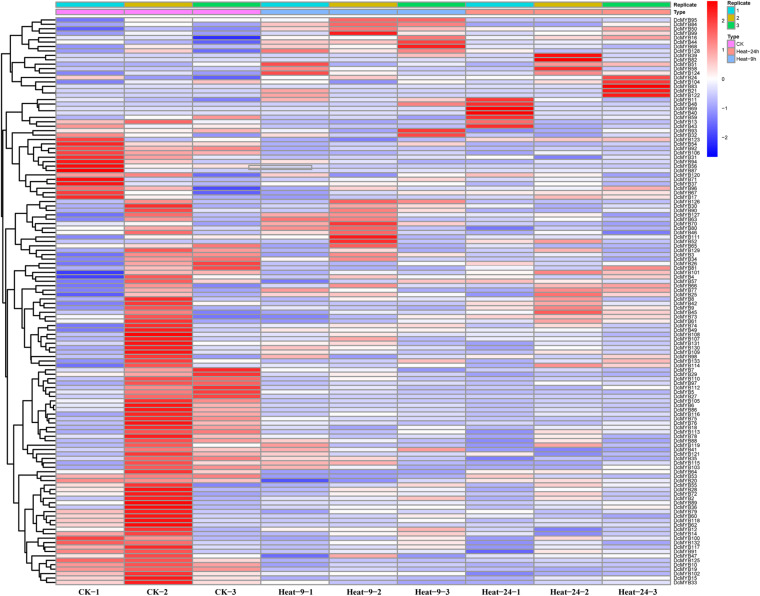
*DcMYB* gene expression in response to heat stress as determined by RNA-seq. Relative expression of different *MYBs* is shown for control and under heat stress after 0, 9, and 24 h. The *Y*-axis shows the gene names and *X*-axis represents the time interval. The different colors correspond to the log2-transformed fold change with blue and red indicating down- and up-regulation, respectively.

**FIGURE 8:**
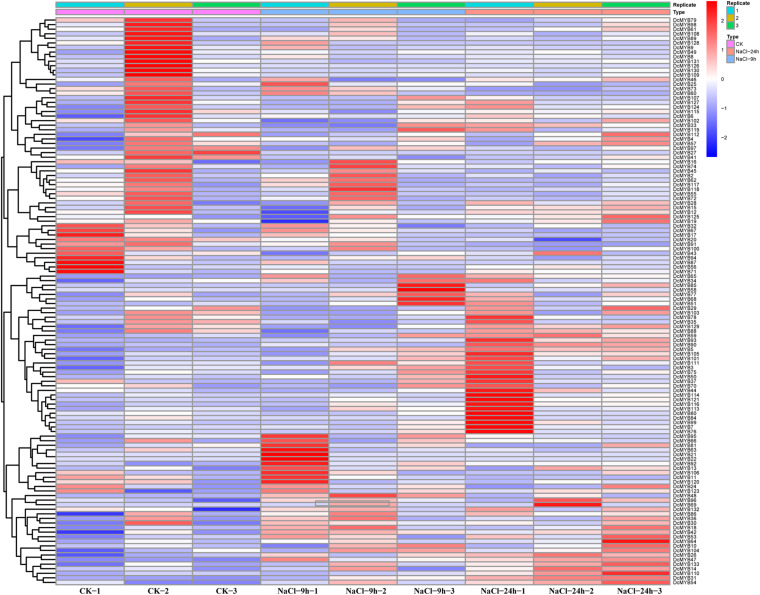
*DcMYB* gene expression in response to cold stress as determined by RNA-seq. Relative expression of different *MYBs* is shown under control and cold stress after 0, 9, and 24 h. The *Y*-axis shows the gene names and *X*-axis represents the time interval. The different colors correspond to the log2-transformed fold change with blue and red indicating down- and up-regulation, respectively.

**FIGURE 9:**
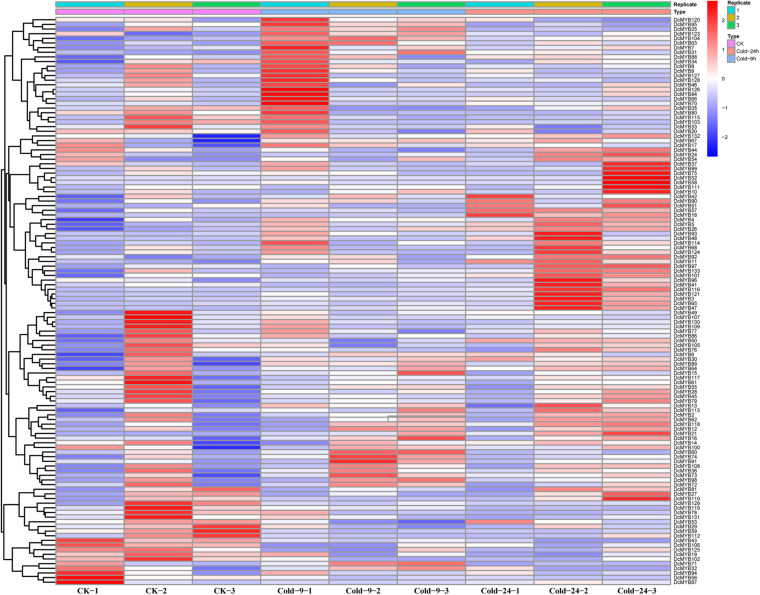
*DcMYB* gene expression in response to salt stress as determined by RNA-seq. Relative expression of different *MYBs* is shown under control and salt stress after 0, 9, and 24 h. The *Y*-axis shows the gene names and *X*-axis represents the time interval. The different colors correspond to the log2-transformed fold change with blue and red indicating down- and up-regulation, respectively.

To investigate the structures of MYB proteins, the conserved motifs were analyzed by TBtools. The width of the ten identified motifs ranged from 8 to 50 amino acids and the maximum E-value of these motifs was 2.4e-124 (Supplementary Figure 2). The motif distribution corresponding to the phylogenetic tree of the DcMYB proteins is shown in Figures 2A,B. High uniformity was found between each subgroup and their types (Supplementary Table 1 and Figure 2A). For example, the members from subgroups A, B, C and D all belonged to the MYB-related family and the members of subgroups F, H, J, K, M and N all belonged to the R2R3-MYB family. Moreover, some specific motifs were identified. The subgroup C and D members only contained motif 10, except DcMYB28 and DcMYB79, which lacked motifs. All the members in subgroup F and H contained motif 7-motif 1-motif 5 in turn, and the subgroup K contained motif 3-motif 8-motif 1-motif 5 in turn. Motif 6 was present only in subgroup A and B. As previously mentioned, the R2R3 subgroup contains the largest number of proteins. In this study, multiple sequence alignments were conducted to investigate features of the homologous R2R3-MYB domain in *D. catenatum*. Subsequently, the WebLogo program was utilized to characterize conserved motifs in R2R3-MYB proteins. The R2 and R3 repeats consist of highly conserved sequences, where 13 out of 54 and 10 out of 49 amino-acids were 100% conserved. These included W-5, E-9, D-10, L-13, G-21, W-26, R-39, K-42, S-43, C-44, R-47, W-48, and N-50 in the R2 repeat (Figure 3A) and E-9, G-21, W-24, R-34, T-35, D-36, N-37, K-40, N-41, and W-43 in the R3 repeat (Figure 3B). Furthermore, R2R3 repeats of the *D. catenatum* R2R3-MYB proteins were found to contain five highly conserved Trp (W) residues, which is consistent with other plant species (He et al., 2016; Zhou Q. et al., 2019), and these W residues play an important role in sequence-specific binding of DNA (Stracke et al., 2001; Wilkins et al., 2009).

The exon/intron structure can provide valuable information concerning evolutionary relationships among taxa. To provide greater insight into the exon/intron structure of the 133 *DcMYB* TFs, the cDNA and corresponding genomic sequences of *D. catenatum* were compared. As shown in Figure 2C, approximately 93.99% of the *DcMYB* genes contained 1 to 12 introns, similar to the *AtMYBs* (Matus et al., 2008). The number of introns in the *DcMYB* genes appeared to be limited, where most *DcMYB* TFs had only 1 (21.05%) or 2 introns (57.14%). The remaining 16.54% contained more than 2 introns in their gene structures. However, there were still 5.26% of genes that lacked introns. *DcMYB17*, belonging to subgroup A, had the most with 16 exons and 15 introns in the gene structure. Remarkably, the members of subgroup C contained more than 5 introns, indicating their introns had been specifically inserted and retained in the genome during evolution of the genes (Brett et al., 2002; Du et al., 2012).

### Promoter Analysis of *MYB* Genes

The *cis*-elements in the promoter region located −2000 to −1 bp upstream of the coding sequences of the *MYB* genes were analyzed against the PlantCARE database. As shown in Figure 4 and Supplementary Table 5, 22 potential functional *cis*-acting elements were detected, which could be divided into three types according to their biological functions. The first type was hormone-related elements containing a salicylic acid responsiveness element (TCA-element and SARE), MeJA-responsiveness element (CGTCA-motif and TGACG-motif), gibberellin-responsive element (GARE-motif, P-box and TATC-box), auxin responsiveness element (AuxRR-core and TGA-element), and abscisic acid responsiveness element (ABRE). The second type was stress-related elements containing a low-temperature responsiveness element (LTR), MYB binding site involved in drought-inducibility (MBS), defense and stress responsiveness element (TC-rich repeats), wound-responsive element (WUN-motif), and anaerobic induction element (ARE). The third type was plant development-related elements, such as endosperm expression element (GCN4_motif), meristem expression element (CAT-box), seed-specific regulation element (RY-element), and element involved in differentiation of the palisade mesophyll cells (HD-Zip 1). Among these, AREs were the most common type of *cis*-element and found in the promoters of 99 of the *DcMYB* genes. Several *cis*-elements were unique to individual *DcMYB* genes, e.g., the SARE was only present in *DcMYB109* and *DcMYB117* and HD-Zip 1 element only in *DcMYB28*, *DcMYB54* and *DcMYB103.*

### Expression Profiles of *MYB* Genes in Different Tissues

To investigate the spatial expression levels of *MYB* genes in *D. catenatum*, the expression profiles of 10 genes from each type across six tissues, roots, stems, leaves, sepals, capsules, and gynostemia, were analyzed by qRT-PCR. As shown in Figure 5 and Supplementary Figure 3, the expression levels of all selected *DcMYB* genes (except *DcMYB3* and *DcMYB53*) were higher in gynostemia than other tested organs. *DcMYB3* was highly expressed in root and the expression of *DcMYB53* did not significantly vary across the different tissues. In this study, *DcMYB108* and *DcMYB72*, belonging to the 3R and 4R family, were highly expressed in gynostemia, suggesting they may play a role in gynostemia dynamics. Interestingly, the 10 genes were all expressed similarly in leaves.

### *MYB* Gene Expression Pattern Analysis Based on RNA-seq

To investigate the expression levels of *DcMYB* genes in response to abiotic stresses, four groups of RNA-seq data, consisting of transcriptome data derived from samples exposed to drought, heat, cold, and salt, were used for analysis. The FPKM of each *MYB* gene in response to the four stresses at the indicated time points was determined based on three replicates and are shown in Supplementary Tables 6–9. Through the RNA-seq data, 123, 128, 122, and 124 *MYB* genes were screened under drought (Figure 6 and Supplementary Table 6), heat (Figure 7 and Supplementary Table 7), cold (Figure 8 and Supplementary Table 8), and salt stress (Figure 9 and Supplementary Table 9), respectively. We screened the genes with absolute log2FC values greater than 1, in which the FC (fold change) was equal to the FPKM of 24 h after treatment divided by the FPKM of no treatment, as differentially expressed genes (DEGs). Based on this, we identified 29 drought-related, 54 heat-related, 46 cold-related and 47 salt-related DEGs. Additionally, under drought stress, 19 and 10 *DcMYB* genes were up- and down-regulated, respectively, after 24 h of treatment. Under heat stress, 4 and 50 *DcMYB* genes were up- and down-regulated, respectively, after 24 h of treatment. Under cold stress, 28 and 18 *DcMYB* genes were up- and down-regulated after 24 h of treatment. Under salinity stress, 31 and 16 *DcMYB* genes were up- and down-regulated after 24 h of treatment. These results indicate that a high number of *DcMYB* genes were responsive to abiotic stresses.

**FIGURE 10:**
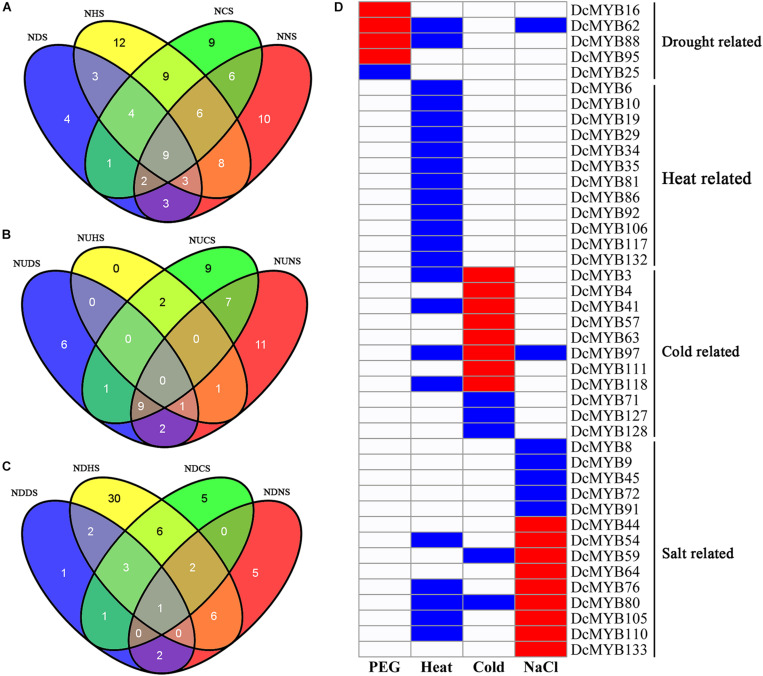
Comparisons of differentially expressed genes and genes with different response patterns following exposure to four stresses. Venn diagrams showing the numbers of differentially expressed genes **(A)** and up- **(B)** and down-regulated genes **(C)** in the four comparisons. ND(H/C/N)S: the number of differentially expressed genes in response to drought (heat/cold/salt) stress; NUD(H/C/N)S: the number of up-regulated genes in response to drought (heat/cold/salt) stress; NDD(H/C/N)S: the number of down-regulated genes in response to drought (heat/cold/salt) stress. **(D)** A representation of a gene specifically expressed, where red, blue, and white indicate up-regulated, down-regulated, and unchanged, respectively.

To investigate their expression patterns under multiple stresses, Venn diagrams of the genes responsive to the four stresses were depicted. As shown in Figure 10A and Supplementary Table 10, many genes were involved in more than one stress response. Nine genes (*DcMYB5*, *DcMYB11*, *DcMYB18*, *DcMYB26*, *DcMYB65*, *DcMYB107*, *DcMYB113*, *DcMYB116*, and *DcMYB121*) participated in all four stress responses, in which *DcMYB107* was down-regulated in all four stresses. Interestingly, *DcMYB51* was up-regulated in response to drought, heat, and salt stresses, suggesting *DcMYB51* might function in response to these stresses. Six and one *DcMYB* genes were up- and down-regulated, respectively, only in response to drought stress. Thirty *DcMYB* genes were down-regulated only under heat stress. Nine and five *DcMYB* genes were up- and down-regulated, respectively, only in response to cold stress. Eleven and five *DcMYB* genes were up- and down-regulated, respectively, only in response to salt stress (Figures 10B,C). The number of up- genes and down-regulated genes involved in the responses to the four stresses differed, indicating the *DcMYB* genes might have different functions in response to stresses.

Additionally, we screened the genes that were differentially expressed under only one stress or whose expression patterns under one stress differed from the other three stresses. A total of 42 genes were screened out. Five, 12, 11, and 14 *DcMYB* genes were specifically related to drought, heat, cold, and salt stress, respectively (Figure 10D).

### Expression Validation of *MYB* Genes via qRT-PCR

**FIGURE 11:**
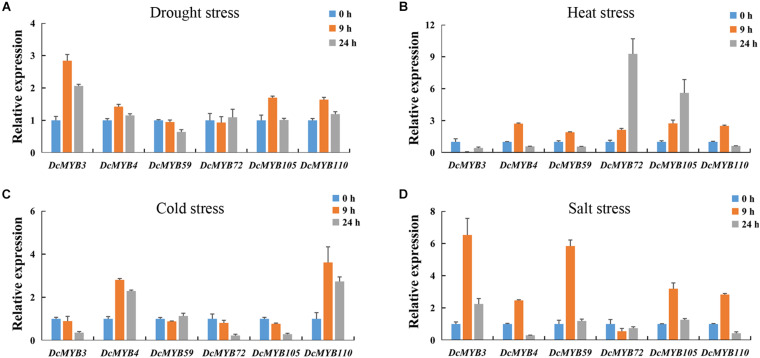
Real-time quantitative PCR validation of six randomly selected genes after 0, 9, or 24 h exposed to one of four stresses. **(A,B)** The expression of the six genes in response to **(A)** drought, **(B)** heat, **(C)** cold, and **(D)** salt stress as measured by qRT-PCR. The standard deviations are shown with error bars.

To validate the accuracy of the expression data obtained from RNA-seq, six MYB genes were selected to study their expression patterns after exposure to the four abiotic stresses through qRT-PCR (Figure 11). The expression levels of the selected genes were generally consistent with the transcriptome data (Figure 10D). This independent evaluation indicates the RNA-seq data were reliable.

## Discussion

MYB transcript factors are common, making up one of the largest TF families in plants, and play important roles in plant growth, development, and responses to abiotic stresses (Dubos et al., 2010; Ruts et al., 2012; Campos et al., 2016). *MYB* genes have been reported in many species, such as *A. thaliana* (Dubos et al., 2010), rice (Katiyar et al., 2012), *Populus trichocarpa* (Cho et al., 2016), grape (Koyama et al., 2014), and *Phalaenopsis equestris* (Wang et al., 2018), in previous studies. However, little is known about *D. catenatum*. Here, we identified 133 MYB members in *D. catenatum* using bioinformatics, analyzed the phylogenetic relationships with *A. thaliana* and rice MYBs, and examined conserved motifs and gene structures, *cis*-elements in the promoters, tissue-specific expression patterns, and expression in response to four abiotic stresses. The number of *MYB* genes in each plant does not completely correlate with the genome size (Sun et al., 2019). Despite the *D. catenatum* genome (1.11 Gb) (Zhang et al., 2016) being larger than that of *A. thaliana* (125 Mb) (Zapata et al., 2016), there were fewer *DcMYB* members than *AtMYB* members (170 members) identified in this study (Figure 1 and Supplementary Table 4). The 133 *DcMYB*s could be classified into 14 subgroups. Subgroup M was the largest group and subgroup H the smallest group. The percentage in each subgroup was similar to *Arabiodpsis* and rice (Supplementary Figure 1), demonstrating that *MYB* genes in different plants have conserved gene duplication. Furthermore, the physical parameters of MYBs in different plants have been shown to be very similar (Zhou Q. et al., 2019; Chen et al., 2021). The MWs of the DcMYB proteins ranged from 10.19 (DcMYB41) to 102.90 kDa (DcMYB118) and the pI values of the predicted proteins from 4.18 (DcMYB117) to 10.63 (DcMYB43) (Supplementary Table 3). The MWs of the potato MYB (*StMYB*) proteins ranged from 5.89 (StMYB26) to 113.39 kDa (StMYB146) and the pI values from to 4.59 (StMYB78) to 10.26 (StMYB10) (Sun et al., 2019), while the MWs of the rice MYB proteins ranged from 7.61 to 109.41 kDa and the pI values from to 3.99 to 12.26 (Katiyar et al., 2012). This suggests the *MYB* gene family is relatively evolutionarily conserved.

In addition, we analyzed the motif compositions of DcMYB proteins (Figure 2). We found most MYB proteins contained motifs 1, 3, 5, and 8. Interestingly, motif 3 (or 7) – motif 8 – motif 1 (or 2) – motif 5 (or motif 4) formed the R2 and R3 repeat (Figure 3 and Supplementary Figure 2). We found the DcMYB proteins containing these motifs were in the R2R3-MYB group, which contained the most MYB members in *D. catenatum* (Supplementary Table 1). Although the motif composition of each subgroup differed, it was similar within each subgroup. For example, subgroups J, K, L, M, and N contained motif 3-motif 8-motif 1-motif 5 and subgroups E, F, H and I contained motif 7-motif 1-motif 5. Some specific motifs were also found in the MYB proteins. Subgroups C and D only contained motifs 10, implying this motif might have a specific function. Gene structures often reflect the evolution of plant gene families (Wang et al., 2013; Liu et al., 2014). To study the intron distribution in the *D. catenatum* genome, we investigated the exon-intron structure of *DcMYB* genes. There were less than 5 introns within subgroups B, F, G, H, I, J, K, L, M, and N, while the number of introns in subgroups A, C, D and E were more variable and irregular (Figure 2). This feature of *MYB* gene structures was similar to the numbers of introns in *Prunus salicina* (Liu C. et al., 2020) and *Physcomitrella patens* (Pu et al., 2020), which suggests intron gain or loss has played important roles in *MYB* evolution.

Tissue differential expression analysis showed all tested *MYB* genes were significantly expressed in *D. catenatum* gynostemia, indicating these genes may have important functions in *D. catenatum* gynostemia development. Genes with high homology in the same branch of a phylogenetic tree generally have high sequence similarity and may also have similar functions (Weston, 1998; Li and Lu, 2014). DcMYB69 belonging to subgroup E had close homology with AtMYB111. A previous study showed AtMYB111 was involved in the biosynthesis of flavanols (Pandey et al., 2014). DcMYB28 and AtMYB59 clustered in the same subgroup, C, in the phylogenetic tree and studies have shown AtMYB59 not only participates in the regulation of cell cycle and root growth, but also plays an important role in the hypokalemic response of *A. thaliana* (Li et al., 2006; Mu et al., 2009). AtMYB52 is involved in the ABA response and enhances the drought tolerance of *Arabidposis* (Park et al., 2011). DcMYB53, DcMYB54, and DcMYB56 in *D. catenatum* and homologous genes of AtMYB52 in *A. thaliana* might be involved in the response to drought stress in an ABA-dependent manner.

As shown in Figure 4 and Supplementary Table 5, most of the *DcMYB* gene promoters have hormone-related *cis*-elements, including a TCA-element, CGTCA-motif, GARE-motif, TGA-element, and ABRE, and abiotic stress-related *cis*-elements, such as LTR, MBS, WUN-motif, and TC-rich repeats. These findings suggest *DcMYB* genes may both directly and indirectly modulate abiotic stress responses in *D. catenatum*. In the present study, expression of some of the *DcMYB* genes significantly changed in response to drought, heat, cold, and/or salt stress as measured by RNA-seq, indicating these genes were involved in resistance to these four stresses in *D. catenatum*. We screened and identified 29, 54, 46, and 47 DEGs responding to drought, heat, cold, and salt, respectively. Only 35 genes were differently expressed in only one type of stresses, indicating most genes are involved in responses to multiple stresses. Many studies have shown certain *MYB* genes have roles in responses to more than one stress. *StMYB3* and *StMYB19* from potato are induced by salt, high temperature, and ABA, IAA, and GA3 treatment (Sun et al., 2019). TaODORANT1, a wheat R2R3-type MYB TF, positively regulates drought and salt tolerance in transgenic tobacco (Wei et al., 2017). The *GrMYB169* gene from *Gossypium raimondii* is induced by drought and salt stress (He et al., 2016). Nine DEGs in this study were involved in responses to all four tested stresses, with only one gene having the same expression pattern in response to all four, indicating most of the DEGs had different expression patterns and functioned in different pathways in response to stress. Of the four stresses, there were the fewest DEGs in response to drought and the most in response to heat. Correspondingly, the drought-specific response genes were also the fewest in number, suggesting *DcMYB* genes may have the weakest response to drought stress.

## Conclusion

In this study, a genome-wide identification of *MYB* genes in *D. catenatum* was performed and a total of 133 *DcMYB* genes were identified. The DcMYB superfamily can be divided into four families, MYB-related, R2R3-MYB, R1R2R3-MYB, and R4-MYB, based on the number of their R motifs. Of these four families, R2R3-MYB had the most members. Members within the same family had similar structures, indicating these proteins might have similar functions. The *MYB* genes were expressed in different tissues, including roots, stems, leaves, capsules, sepals, and gynostemia, and some displayed tissue-specific expression patterns. Analysis of gene expression in responses to abiotic stresses by RNA-seq and qRT-PCR revealed MYB families have wide expression profiles and play key roles in stress responses. Our results provide a basis for identifying important candidate *DcMYB* genes involved in responses to abiotic stresses.

## Data Availability Statement

The original contributions presented in the study are included in the article/Supplementary Material, further inquiries can be directed to the corresponding authors.

## Author Contributions

YZ and JW conceived and designed the study and prepared the manuscript. TZ, ZC, YL, and YK performed the experiments. TZ, ZC, and XS assisted with the analysis and interpretation of the data. YZ and TZ drafted the manuscript. YZ participated in the design of the experiments and provided a critical review. All authors have read, edited, and approved the current version of the manuscript.

## Conflict of Interest

The authors declare that the research was conducted in the absence of any commercial or financial relationships that could be construed as a potential conflict of interest.

## Publisher’s Note

All claims expressed in this article are solely those of the authors and do not necessarily represent those of their affiliated organizations, or those of the publisher, the editors and the reviewers. Any product that may be evaluated in this article, or claim that may be made by its manufacturer, is not guaranteed or endorsed by the publisher.

## Footnotes

1
http://pfam.xfam.org/

2
https://www.ncbi.nlm.nih.gov/cdd/

3
http://smart.embl.de/smart/batch.pl

4
http://www.cbs.dtu.dk/services/TMHMM/

5
https://www.genscript.com/psort.html

6
https://evolgenius.info/evolview-v2

7
http://weblogo.berkeley.edu/logo.cgi

8
http://bioinformatics.psb.ugent.be/webtools/plantcare/html/

9
https://www.ncbi.nlm.nih.gov/search/all/?term=PRJNA262478
