# Supplementary figures and images for: Genome-Wide Identification and Expression Analysis of MYB Transcription Factor Superfamily in Dendrobium catenatum
Source: Front Genet. 2021 Aug 26;12:714696. doi: 10.3389/fgene.2021.714696 (PMC8427673; doi:10.3389/fgene.2021.714696)

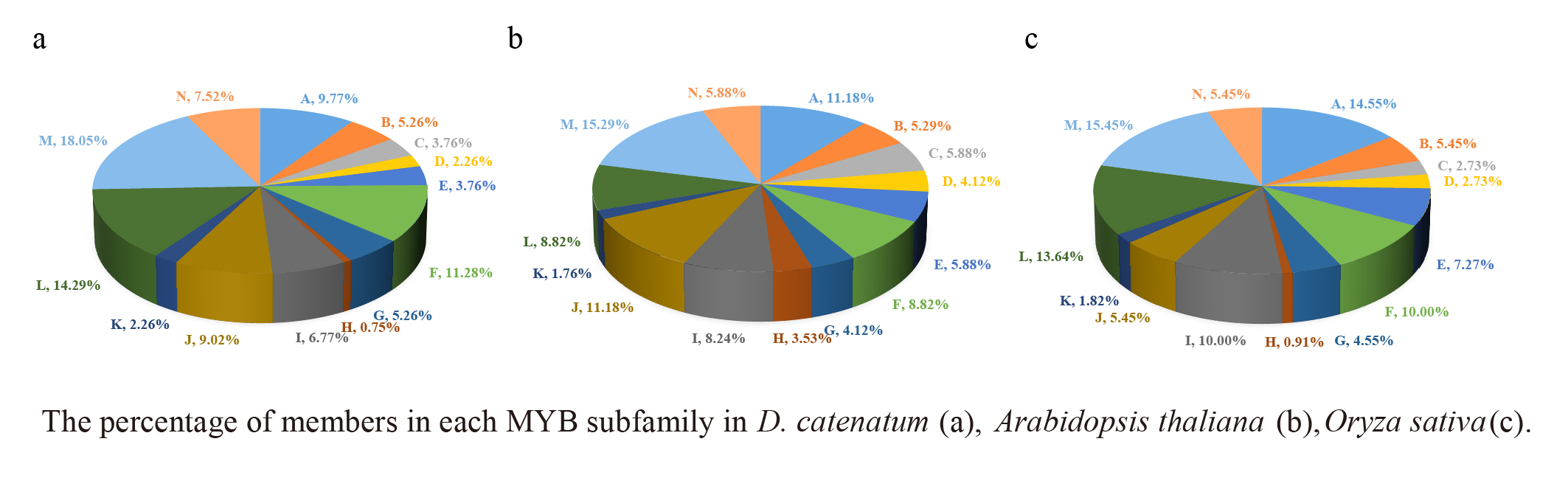

Supplement: Supplementary file 1 [file Image_1.TIF]

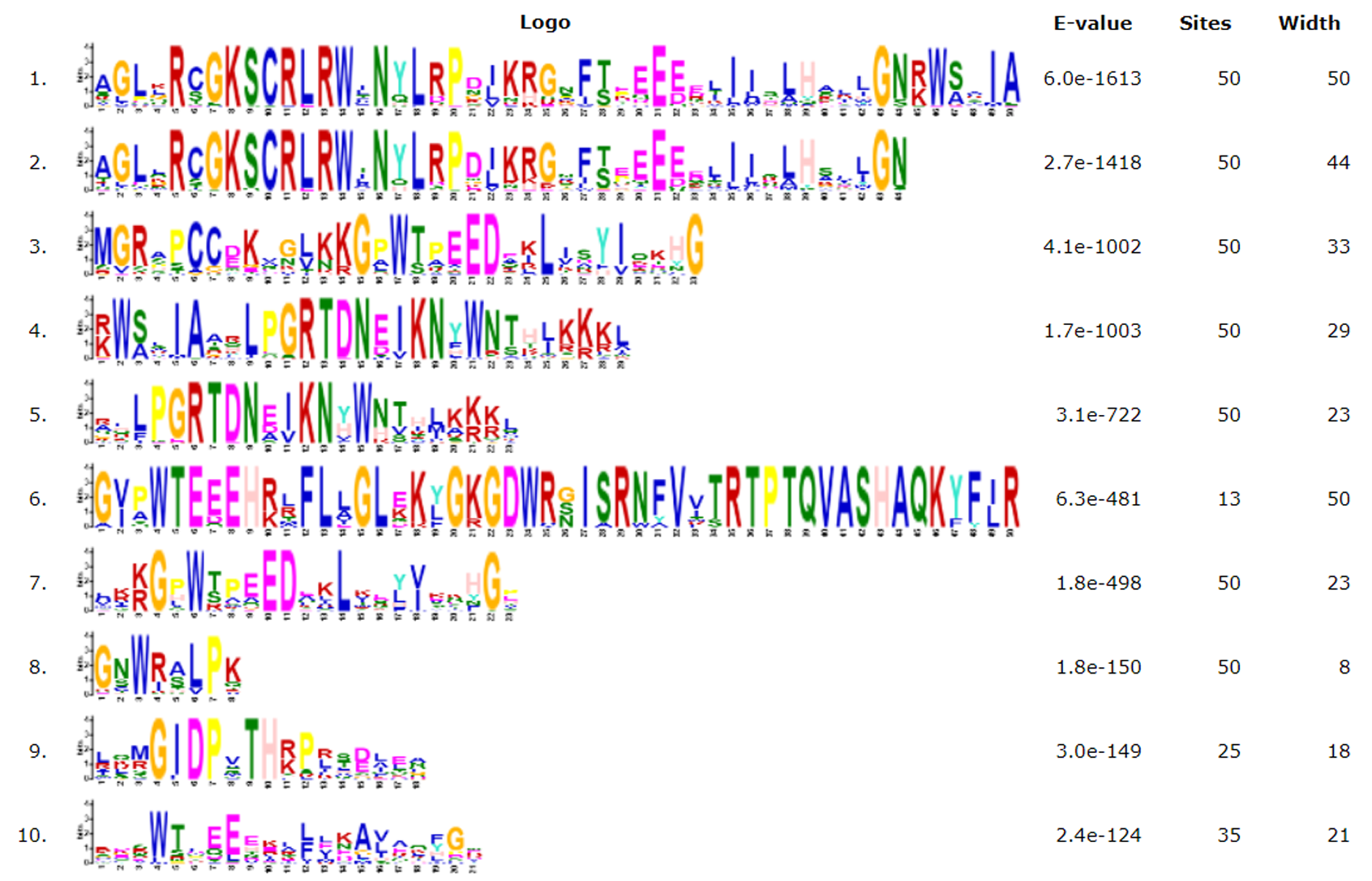

Supplement: Supplementary file 2 [file Image_2.TIF]

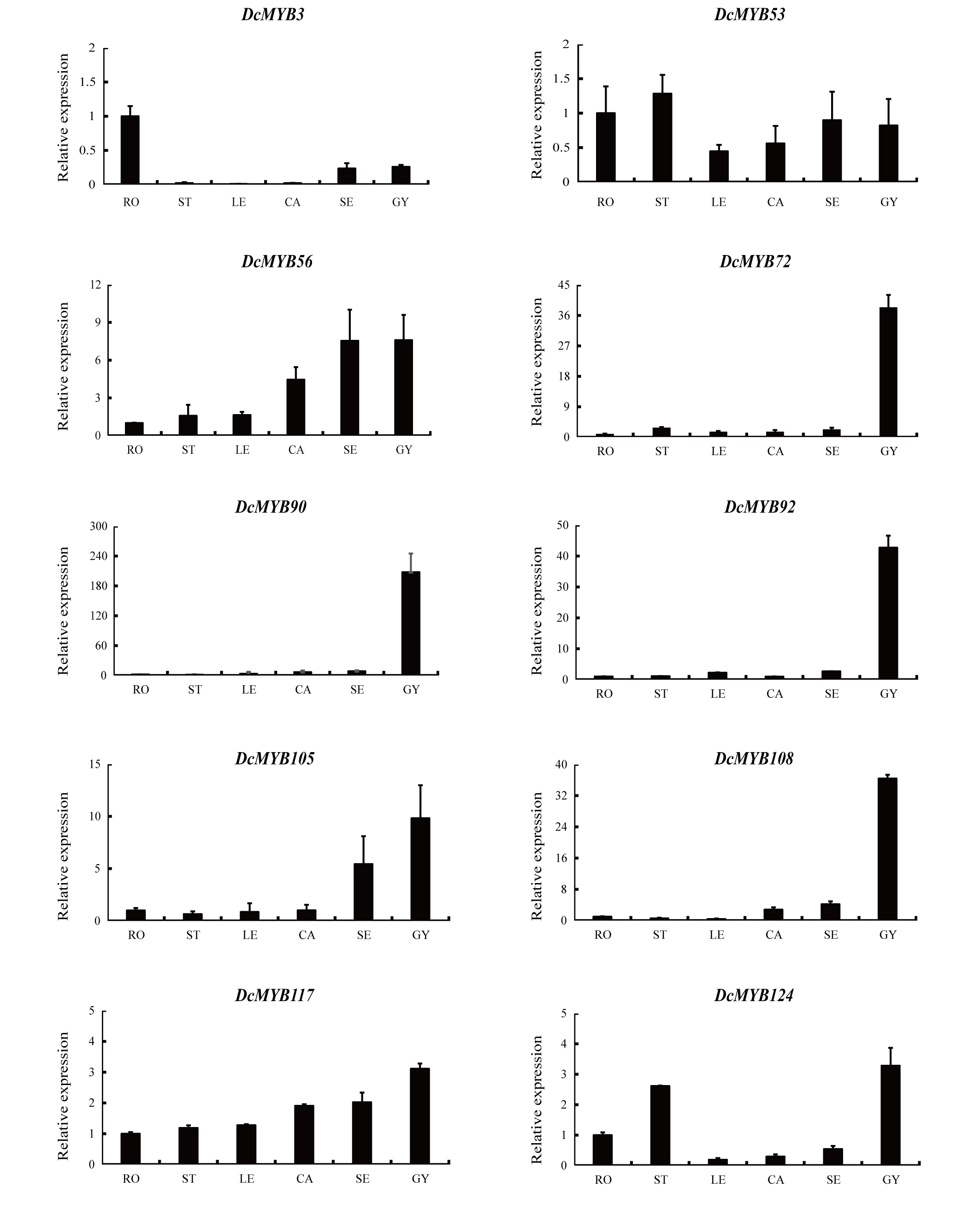

Supplement: Supplementary file 3 [file Image_3.TIF]
