# Supplementary material for: Genome-Wide Identification and Expression Analysis of MYB Transcription Factor Superfamily in Dendrobium catenatum
Source: Front Genet. 2021 Aug 26;12:714696. doi: 10.3389/fgene.2021.714696 (PMC8427673; doi:10.3389/fgene.2021.714696)
Supplement: Supplementary file 4 [file Data_Sheet_1.zip › Table 1.docx]

**TABLE S1** Physicochemical properties of *Dendrobium catenatum* MYB proteins.

| Gene name | Gene ID | Locus | Length | MW(kDa) | pI | No. of Transmembrane | Subcellular localization | Type |
| --- | --- | --- | --- | --- | --- | --- | --- | --- |
| DcMYB1 | LOC110112960 | NW_021319446.1:189320..193537 | 233 | 26.23 | 8.29 | no | Nuclear | MYB-related |
| DcMYB2 | LOC110098636 | NW_021319551.1:1485089..1517971 | 776 | 85.51 | 6.44 | no | Nuclear | MYB-related |
| DcMYB3 | LOC110111177 | NW_021320019.1:4940077..4942214 | 227 | 26.16 | 5.98 | no | Nuclear | R2R3-MYB |
| DcMYB4 | LOC110098229 | NW_021318839.1:236764..238891 | 227 | 25.71 | 4.92 | no | Nuclear | MYB-related |
| DcMYB5 | LOC110094080 | NW_021318618.1:3039665..3041446 | 312 | 35.18 | 7.46 | no | Nuclear | R2R3-MYB |
| DcMYB6 | LOC110093341 | NW_021319120.1:150908..153402 | 373 | 41.85 | 6.17 | no | Nuclear | R2R3-MYB |
| DcMYB7 | LOC110092191 | NW_021319862.1:879035..880434 | 233 | 25.87 | 7.88 | no | Nuclear | R2R3-MYB |
| DcMYB8 | LOC110113232 | NW_021463285.1:757894..759901 | 409 | 45.59 | 7.52 | no | Nuclear | R2R3-MYB |
| DcMYB9 | LOC110115321 | NW_021525030.1:2027..3724 | 409 | 45.60 | 7.52 | no | Nuclear | R2R3-MYB |
| DcMYB10 | LOC110100853 | NW_021319733.1:114778..117068 | 203 | 23.36 | 8.33 | no | Nuclear | R2R3-MYB |
| DcMYB11 | LOC110105011 | NW_021319518.1:7928576..7933475 | 307 | 34.92 | 4.85 | no | Nuclear | R2R3-MYB |
| DcMYB12 | LOC110104349 | NW_021319682.1:2829464..2833756 | 288 | 30.47 | 7.32 | no | Nuclear | MYB-related |
| DcMYB13 | LOC110103608 | NW_021319394.1:38702..48875 | 190 | 21.34 | 9.92 | no | Nuclear | MYB-related |
| DcMYB14 | LOC110112704 | NW_021319089.1:1217759..1219090 | 255 | 28.18 | 7.18 | no | Nuclear | R2R3-MYB |
| DcMYB15 | LOC110095967 | NW_021318785.1:1177013..1182095 | 174 | 19.67 | 8.78 | no | Nuclear | R2R3-MYB |
| DcMYB16 | LOC110098894 | NW_021319843.1:443267..447865 | 284 | 30.46 | 8.93 | no | Nuclear | MYB-related |
| DcMYB17 | LOC110112338 | NW_021319083.1:18582993..18614675 | 819 | 91.24 | 7.06 | no | Nuclear | MYB-related |
| DcMYB18 | LOC110106750 | NW_021319879.1:289947..293525 | 454 | 49.81 | 5.93 | no | Nuclear | MYB-related |
| DcMYB19 | LOC110110250 | NW_021319202.1:503555..518577 | 576 | 64.39 | 7.98 | no | Nuclear | MYB-related |
| DcMYB20 | LOC110115620 | NW_021318726.1:1047430..1065150 | 370 | 40.22 | 6.76 | no | Nuclear | R2R3-MYB |
| DcMYB21 | LOC110092557 | NW_021318852.1:1571344..1577134 | 290 | 32.70 | 4.95 | no | Nuclear | R2R3-MYB |
| DcMYB22 | LOC110092560 | NW_021318852.1:1546401..1554455 | 174 | 19.41 | 5.24 | no | Nuclear | MYB-related |
| DcMYB23 | LOC110106773 | NW_021319810.1:255545..258456 | 254 | 29.16 | 7.34 | no | Nuclear | R2R3-MYB |
| DcMYB24 | LOC110098032 | NW_021319188.1:691972..693962 | 203 | 23.54 | 7.98 | no | Nuclear | R2R3-MYB |
| DcMYB25 | LOC110113344 | NW_021319518.1:5720043..5724206 | 316 | 35.29 | 5.56 | no | Nuclear | R2R3-MYB |
| DcMYB26 | LOC110100661 | NW_021319120.1:565151..566139 | 243 | 26.20 | 8.34 | no | Nuclear | R2R3-MYB |
| DcMYB27 | LOC110098178 | NW_021318693.1:3922890..3923450 | 186 | 20.76 | 9.34 | no | Nuclear | R2R3-MYB |
| DcMYB28 | LOC110104044 | NW_021319746.1:1630588..1644081 | 289 | 32.09 | 10.16 | no | Nuclear | MYB-related |
| DcMYB29 | LOC110096293 | NW_021318648.1:652127..653832 | 298 | 32.99 | 8.72 | no | Nuclear | R2R3-MYB |
| DcMYB30 | LOC110099604 | NW_021320177.1:103592..107640 | 330 | 37.49 | 8.13 | no | Nuclear | R2R3-MYB |
| DcMYB31 | LOC110110539 | NW_021319360.1:1275282..1277910 | 357 | 40.08 | 7.48 | no | Nuclear | R2R3-MYB |
| DcMYB32 | LOC110116384 | NW_021319179.1:132477..135870 | 303 | 33.12 | 5.65 | no | Nuclear | R2R3-MYB |
| DcMYB33 | LOC110097361 | NW_021319860.1:1382698..1385207 | 291 | 33.07 | 8.79 | no | Nuclear | R2R3-MYB |
| DcMYB34 | LOC110111576 | NW_021318629.1:181884..183737 | 243 | 27.92 | 8.97 | no | Nuclear | R2R3-MYB |
| DcMYB35 | LOC110105411 | NW_021319584.1:88750..90163 | 286 | 32.20 | 5.26 | no | Nuclear | R2R3-MYB |
| DcMYB36 | LOC110098841 | NW_021319132.1:1142491..1171694 | 530 | 60.52 | 9.16 | no | Nuclear | MYB-related |
| DcMYB37 | LOC110107270 | NW_021318595.1:5714386..5724516 | 326 | 35.85 | 6.36 | no | Nuclear | R2R3-MYB |
| DcMYB38 | LOC110104578 | NW_021451214.1:773..1549 | 104 | 11.91 | 10.5 | no | Nuclear | R2R3-MYB |
| DcMYB39 | LOC110111434 | NW_021319876.1:552953..568197 | 213 | 24.55 | 10.54 | no | Nuclear | R2R3-MYB |
| DcMYB40 | LOC110102852 | NW_021318711.1:1341878..1344747 | 235 | 27.03 | 5.10 | no | Nuclear | R2R3-MYB |
| DcMYB41 | LOC110102742 | NW_021320019.1:6754339..6755346 | 85 | 10.19 | 10.34 | no | Cytoplasmic | MYB-related |
| DcMYB42 | LOC110109430 | NW_021318999.1:155432..157225 | 238 | 26.55 | 8.07 | no | Nuclear | R2R3-MYB |
| DcMYB43 | LOC110111176 | NW_021320019.1:5175202..5190993 | 210 | 23.15 | 10.63 | no | Nuclear | MYB-related |
| DcMYB44 | LOC110103101 | NW_021320146.1:886979..888249 | 258 | 28.57 | 7.02 | no | Nuclear | R2R3-MYB |
| DcMYB45 | LOC110093354 | NW_021319184.1:658608..788518 | 406 | 45.59 | 6.52 | no | Mitochondrial | MYB-related |
| DcMYB46 | LOC110098041 | NW_021319188.1:133396..134934 | 283 | 31.49 | 8.38 | no | Nuclear | R2R3-MYB |
| DcMYB47 | LOC110101288 | NW_021319682.1:9371306..9372968 | 247 | 28.19 | 4.66 | no | Nuclear | R2R3-MYB |
| DcMYB48 | LOC110096958 | NW_021417401.1:153046..154705 | 242 | 27.78 | 5.42 | no | Nuclear | R2R3-MYB |
| DcMYB49 | LOC110107869 | NW_021319309.1:5247884..5249793 | 309 | 33.73 | 5.40 | no | Nuclear | R2R3-MYB |
| DcMYB50 | LOC110099019 | NW_021318618.1:7860011..7861547 | 238 | 28.03 | 6.06 | no | Nuclear | R2R3-MYB |
| DcMYB51 | LOC110097914 | NW_021320182.1:602133..604918 | 272 | 30.68 | 6.06 | no | Nuclear | R2R3-MYB |
| DcMYB52 | LOC110104286 | NW_021320055.1:571475..574570 | 222 | 25.79 | 6.92 | no | Nuclear | R2R3-MYB |
| DcMYB53 | LOC110108441 | NW_021319683.1:1203407..1205726 | 295 | 33.26 | 7.99 | no | Nuclear | MYB-related |
| DcMYB54 | LOC110114193 | NW_021319178.1:24949602..24952214 | 295 | 33.08 | 7.03 | no | Nuclear | MYB-related |
| DcMYB55 | LOC110109995 | NW_021320138.1:111695..132298 | 578 | 65.33 | 6.63 | no | Nuclear | MYB-related |
| DcMYB56 | LOC110094156 | NW_021318533.1:2238716..2240740 | 289 | 32.37 | 7.32 | no | Nuclear | MYB-related |
| DcMYB57 | LOC110099051 | NW_021320094.1:1187696..1189079 | 331 | 36.66 | 6.01 | no | Nuclear | R2R3-MYB |
| DcMYB58 | LOC110104416 | NW_021318566.1:594232..597114 | 322 | 36.54 | 4.60 | no | Cytoplasmic | R2R3-MYB |
| DcMYB59 | LOC110109864 | NW_021318478.1:19802..20705 | 220 | 24.95 | 7.08 | no | Nuclear | MYB-related |
| DcMYB60 | LOC110109300 | NW_021319862.1:1904663..1906670 | 361 | 39.40 | 7.22 | no | Nuclear | R2R3-MYB |
| DcMYB61 | LOC110115888 | NW_021318785.1:2913759..2918148 | 225 | 25.60 | 9.94 | no | Nuclear | MYB-related |
| DcMYB62 | LOC110103760 | NW_021319178.1:1131639..1133915 | 241 | 27.32 | 9.34 | no | Nuclear | R2R3-MYB |
| DcMYB63 | LOC110113544 | NW_021320019.1:9113857..9115526 | 272 | 31.11 | 6.50 | no | Nuclear | R2R3-MYB |
| DcMYB64 | LOC110115927 | NW_021319683.1:693905..695699 | 340 | 38.34 | 7.89 | no | Nuclear | R2R3-MYB |
| DcMYB65 | LOC110096945 | NW_021318796.1:312432..314085 | 232 | 26.15 | 4.74 | no | Nuclear | R2R3-MYB |
| DcMYB66 | LOC110111024 | NW_021319594.1:30768..32848 | 326 | 37.06 | 5.11 | no | Nuclear | R2R3-MYB |
| DcMYB67 | LOC110108439 | NW_021319683.1:1462422..1495699 | 429 | 46.96 | 7.19 | no | Nuclear | MYB-related |
| DcMYB68 | LOC110092042 | NW_021318710.1:26614..27479 | 231 | 27.51 | 7.55 | no | Nuclear | R2R3-MYB |
| DcMYB69 | LOC110102535 | NW_021319394.1:292852..295337 | 286 | 32.57 | 8.23 | no | Nuclear | R2R3-MYB |
| DcMYB70 | LOC110113498 | NW_021318735.1:1023542..1025337 | 326 | 37.38 | 6.84 | no | Nuclear | R2R3-MYB |
| DcMYB71 | LOC110104655 | NW_021319518.1:7509534..7511409 | 327 | 36.77 | 6.85 | no | Nuclear | R2R3-MYB |
| DcMYB72 | LOC110114505 | NW_021320070.1:3046069..3051141 | 714 | 78.78 | 6.18 | no | Nuclear | 4R-MYB |
| DcMYB73 | LOC110103727 | NW_021318492.1:672857..682671 | 267 | 29.18 | 6.31 | no | Nuclear | MYB-related |
| DcMYB74 | LOC110106196 | NW_021318728.1:204563..205627 | 227 | 25.52 | 8.35 | no | Nuclear | R2R3-MYB |
| DcMYB75 | LOC110096046 | NW_021319690.1:293244..295252 | 324 | 36.49 | 7.50 | no | Nuclear | R2R3-MYB |
| DcMYB76 | LOC110114685 | NW_021319083.1:15998768..16000470 | 337 | 37.32 | 7.59 | no | Nuclear | R2R3-MYB |
| DcMYB77 | LOC110105399 | NW_021319584.1:222903..225047 | 330 | 36.77 | 6.70 | no | Nuclear | R2R3-MYB |
| DcMYB78 | LOC110103484 | NW_021319214.1:133944..136273 | 221 | 24.56 | 10.5 | no | Mitochondrial | MYB-related |
| DcMYB79 | LOC110108615 | NW_021503689.1:622428..646749 | 318 | 35.19 | 9.23 | no | Nuclear | MYB-related |
| DcMYB80 | LOC110095224 | NW_021318787.1:1788546..1790349 | 317 | 35.62 | 7.73 | no | Nuclear | R2R3-MYB |
| DcMYB81 | LOC110104906 | NW_021320142.1:216075..219149 | 205 | 23.18 | 5.72 | no | Nuclear | R2R3-MYB |
| DcMYB82 | LOC110113694 | NW_021319086.1:875719..878674 | 276 | 31.46 | 9.84 | no | Nuclear | R2R3-MYB |
| DcMYB83 | LOC110105078 | NW_021319486.1:2305867..2309781 | 200 | 23.01 | 5.72 | no | Nuclear | R2R3-MYB |
| DcMYB84 | LOC110114027 | NW_021513367.1:149965..151663 | 311 | 35.50 | 6.52 | no | Nuclear | R2R3-MYB |
| DcMYB85 | LOC110103895 | NW_021319436.1:3122651..3139918 | 289 | 31.90 | 6.94 | no | Cytoplasmic | R2R3-MYB |
| DcMYB86 | LOC110114551 | NW_021318619.1:1856078..1859030 | 314 | 35.14 | 6.94 | no | Nuclear | R2R3-MYB |
| DcMYB87 | LOC110112113 | NW_021320149.1:166142..168230 | 298 | 33.58 | 7.1 | no | Nuclear | R2R3-MYB |
| DcMYB88 | LOC110108672 | NW_021318516.1:2009255..2011290 | 352 | 39.17 | 7.07 | no | Nuclear | R2R3-MYB |
| DcMYB89 | LOC110095044 | NW_021319797.1:37867..42062 | 165 | 18.99 | 4.65 | no | Nuclear | MYB-related |
| DcMYB90 | LOC110115301 | NW_021319048.1:170278..171649 | 297 | 33.67 | 4.99 | no | Nuclear | R2R3-MYB |
| DcMYB91 | LOC110108743 | NW_021600483.1:128048..129871 | 216 | 24.04 | 9.49 | no | Nuclear | R2R3-MYB |
| DcMYB92 | LOC110096210 | NW_021319690.1:985989..987346 | 316 | 34.60 | 9.80 | no | Nuclear | R2R3-MYB |
| DcMYB93 | LOC110094865 | NW_021525301.1:735..1479 | 164 | 19.03 | 10.61 | no | Nuclear | R2R3-MYB |
| DcMYB94 | LOC110092846 | NW_021395514.1:287..1238 | 88 | 10.41 | 10.58 | no | Nuclear | R2R3-MYB |
| DcMYB95 | LOC110104863 | NW_021319694.1:1916568..1918848 | 244 | 27.79 | 7.01 | no | Nuclear | R2R3-MYB |
| DcMYB96 | LOC110094774 | NW_021319121.1:606910..608917 | 275 | 30.98 | 6.83 | no | Nuclear | R2R3-MYB |
| DcMYB97 | LOC110114571 | NW_021319136.1:132034..133521 | 242 | 27.11 | 4.69 | no | Nuclear | R2R3-MYB |
| DcMYB98 | LOC110112528 | NW_021319259.1:68218..69211 | 258 | 29.64 | 9.87 | no | Nuclear | R2R3-MYB |
| DcMYB99 | LOC110110329 | NW_021319098.1:577787..579426 | 317 | 35.66 | 8.88 | no | Nuclear | R2R3-MYB |
| DcMYB100 | LOC110116648 | NW_021319567.1:240499..266784 | 264 | 29.84 | 6.22 | 2 | Endoplasmic reticulum | MYB-related |
| DcMYB101 | LOC110112193 | NW_021318640.1:537069..538299 | 261 | 29.80 | 9.14 | no | Nuclear | R2R3-MYB |
| DcMYB102 | LOC110112067 | NW_021318636.1:206260..207986 | 231 | 26.79 | 10.11 | no | Nuclear | R2R3-MYB |
| DcMYB103 | LOC110098306 | NW_021319551.1:102287..104194 | 224 | 26.08 | 7.71 | no | Nuclear | R2R3-MYB |
| DcMYB104 | LOC110107128 | NW_021319910.1:335806..337032 | 250 | 28.15 | 6.71 | no | Nuclear | R2R3-MYB |
| DcMYB105 | LOC110099272 | NW_021319480.1:175724..177305 | 280 | 31.25 | 6.51 | no | Nuclear | R2R3-MYB |
| DcMYB106 | LOC110113595 | NW_021320019.1:8788056..8790485 | 293 | 31.25 | 9.28 | no | Nuclear | MYB-related |
| DcMYB107 | LOC110107258 | NW_021318505.1:1656701..1657525 | 241 | 27.95 | 6.79 | no | Nuclear | R2R3-MYB |
| DcMYB108 | LOC110091872 | NW_021319588.1:633391..659772 | 677 | 74.12 | 6.6 | no | Nuclear | 3R-MYB |
| DcMYB109 | LOC110099916 | NW_021318796.1:2733421..2747622 | 291 | 33.55 | 9.08 | no | Nuclear | R2R3-MYB |
| DcMYB110 | LOC110110656 | NW_021320101.1:841414..843035 | 261 | 30.00 | 7.97 | no | Nuclear | R2R3-MYB |
| DcMYB111 | LOC110094698 | NW_021318705.1:1010027..1012312 | 264 | 30.32 | 8.46 | no | Nuclear | R2R3-MYB |
| DcMYB112 | LOC110111286 | NW_021319627.1:325826..328102 | 273 | 31.05 | 9.39 | no | Nuclear | R2R3-MYB |
| DcMYB113 | LOC110110823 | NW_021319095.1:289306..291209 | 321 | 36.81 | 8.56 | no | Nuclear | R2R3-MYB |
| DcMYB114 | LOC110110809 | NW_021319095.1:313244..314806 | 312 | 35.75 | 7.78 | no | Nuclear | R2R3-MYB |
| DcMYB115 | LOC110093022 | NW_021391528.1:263936..265693 | 277 | 31.59 | 6.12 | no | Nuclear | R2R3-MYB |
| DcMYB116 | LOC110098979 | NW_021318864.1:805933..807584 | 336 | 38.19 | 5.36 | no | Nuclear | R2R3-MYB |
| DcMYB117 | LOC110105181 | NW_021319367.1:514838..529908 | 626 | 69.44 | 4.18 | no | Mitochondrial | MYB-related |
| DcMYB118 | LOC110099534 | NW_021380961.1:508676..515334 | 903 | 102.9 | 6.95 | no | Nuclear | MYB-related |
| DcMYB119 | LOC110111728 | NW_021319455.1:235529..236277 | 166 | 19.68 | 10.03 | no | Nuclear | R2R3-MYB |
| DcMYB120 | LOC110108449 | NW_021319683.1:1500947..1504938 | 320 | 37.12 | 9.88 | no | Nuclear | R2R3-MYB |
| DcMYB121 | LOC110110815 | NW_021319095.1:305720..307018 | 292 | 33.14 | 6.16 | no | Nuclear | R2R3-MYB |
| DcMYB122 | LOC110107979 | NW_021319178.1:16740570..16744216 | 389 | 43.65 | 6.20 | no | Nuclear | R2R3-MYB |
| DcMYB123 | LOC110114678 | NW_021319136.1:772697..773756 | 211 | 23.97 | 8.76 | no | Nuclear | R2R3-MYB |
| DcMYB124 | LOC110112867 | NW_021345501.1:966044..966989 | 251 | 28.69 | 8.43 | no | Nuclear | R2R3-MYB |
| DcMYB125 | LOC110099624 | NW_021318709.1:62149..63648 | 207 | 23.86 | 8.41 | no | Nuclear | R2R3-MYB |
| DcMYB126 | LOC110113875 | NW_021318827.1:507138..508283 | 310 | 35.46 | 7.41 | no | Nuclear | R2R3-MYB |
| DcMYB127 | LOC110107882 | NW_021319309.1:5003939..5005850 | 348 | 38.97 | 7.88 | no | Nuclear | R2R3-MYB |
| DcMYB128 | LOC110108407 | NW_021319952.1:334682..335649 | 253 | 29.24 | 10.21 | no | Nuclear | R2R3-MYB |
| DcMYB129 | LOC110105777 | NW_021319804.1:24909..26639 | 237 | 27.28 | 6.67 | no | Nuclear | R2R3-MYB |
| DcMYB130 | LOC110094240 | NW_021318547.1:325349..326948 | 292 | 32.53 | 6.64 | no | Nuclear | R2R3-MYB |
| DcMYB131 | LOC110105838 | NW_021319478.1:764558..767474 | 287 | 32.41 | 6.42 | no | Nuclear | R2R3-MYB |
| DcMYB132 | LOC110110742 | NW_021318796.1:2334738..2339627 | 651 | 74.12 | 6.99 | no | Nuclear | MYB-related |
| DcMYB133 | LOC110107768 | NW_021318852.1:3858649..3862328 | 301 | 32.90 | 9.93 | no | Nuclear | MYB-related |
